# Long non-coding RNA TUSC8 inhibits breast cancer growth and metastasis via miR-190b-5p/MYLIP axis

**DOI:** 10.18632/aging.102791

**Published:** 2020-02-09

**Authors:** Luqing Zhao, Yangying Zhou, Yuelong Zhao, Qingling Li, Jianhua Zhou, Yitao Mao

**Affiliations:** 1 Department of Pathology, Xiangya Hospital, Central South University, Changsha 410008, Hunan, China; 2 Department of Pathology, School of Basic Medical Science, Xiangya School of Medicine, Central South University, Changsha 410013, Hunan, China; 3 Department of Oncology, Xiangya Hospital, Central South University, Changsha 410008, Hunan, China; 4 School of Computer Science and Engineering, South China University of Technology, Guangzhou 510640, Guangdomg, China; 5 Department of Radiology, Xiangya Hospital, Central South University, Changsha 410008, Hunan, China; 6 National Clinical Research Center for Geriatric Disorders, Xiangya Hospital, Central South University, Changsha 410008, Hunan, China

**Keywords:** TUSC8, ceRNA, MYLIP, breast cancer, metastasis

## Abstract

The lncRNA tumor suppressor candidate 8 (TUSC8) plays a critical role in the development of several cancers. However, the biological functions and underlying molecular mechanisms of TUSC8 with respect to breast cancer remain largely unclear. Here, we found that TUSC8 was significantly down-regulated in breast cancer tissues and its high expression predicted better prognosis of breast cancer patients. Functionally, knock-down of TUSC8 drastically promoted the proliferation, migration and invasion of breast cancer cells *in vitro* and facilitated tumorigenicity and metastasis *in vivo*. Mechanistically, the results of luciferase reporter, RIP and RNA pull-down assays proved that TUSC8 functioned as molecular sponge for miR-190b-5p. Furthermore, we showed that TUSC8 served as a competing endogenous RNA (ceRNA) of myosin regulatory light chain interacting protein (MYLIP) through competitively binding with miR-190b-5p and suppressed breast cancer metastasis through regulating the expression of epithelial–mesenchymal transition (EMT) related markers. Clinically, the receiver operating characteristic curve (ROC) analyses revealed that the combination usage of TUSC8 and MYLIP might become novel promising diagnostic biomarkers for breast cancer. Taken together, these results suggested that TUSC8 inhibited breast cancer growth and metastasis via miR-190b-5p/MYLIP axis, providing us new insights into developing potential therapeutic targets for breast cancer patients.

## INTRODUCTION

Breast cancer is the most common and aggressive malignancy occurring in women worldwide. Although the therapeutic advances in diagnosis and clinical treatment for breast cancer have been achieved, the distant metastasis and chemo-resistance are the leading causes of patient recurrence and death [1, 2]. As breast cancer can be categorized into various subtypes according to histopathological parameters or genomic signatures, and different subtypes exhibit varieties of cancer incidence, therapeutic response and prognosis [3–5], suggesting that it is of paramount importance to identify efficient diagnostic biomarkers and therapeutic targets for breast cancer.

Long non-coding RNAs (lncRNAs) are a cluster of non-coding RNA transcripts longer than 200 nucleotides in length and without protein-coding potential. Numerous studies have demonstrated that lncRNAs play essential roles in cancer development processes, mainly including cell growth, apoptosis, autophagy, angiogenesis, stemness, cancer metabolism, immune response, metastasis, chemo-resistance and so on [6–11]. The lncRNA tumor suppressor candidate 8 (TUSC8), also named as XLOC_010588 and LINC01071, is located on chromosome 13q14.11 and is a non-protein coding transcript. Previous studies have shown that over-expression of TUSC8 could inhibit the invasion and migration of cervical cancer cells by up-regulating PTEN via miR-641 [12]. Low expression of long non-coding XLOC_010588 indicated a poor prognosis and it played a pivotal role in cervical cancer cell proliferation via decreasing c-Myc expression [13]. However, XLOC_010588 might function as an oncogene in colorectal cancer. Down-regulation of XLOC_010588 inhibited the invasion and migration of colorectal cancer cells through regulating genes associated with EMT [14]. Furthermore, TUSC8 (LINC01071) showed consistent down-regulation in gastric cancer (GC) compared with adjacent non-tumor tissues and was significantly correlated with the age and gender of the GC patients, respectively [15, 16]. However, apart from the above studies, the biological functions and underlying molecular mechanisms of TUSC8 with respect to breast cancer remain largely unclear.

In this study, we focused on investigating the functional roles of TUSC8 in breast cancer development. We demonstrated that TUSC8 inhibited tumor growth and metastasis of breast cancer cells through sponging miR-190b-5p, leading to the down-regulation of MYLIP. Thus, we reported that a novel regulatory pathway composed of TUSC8/miR-190b-5p/ MYLIP was involved in the progression of breast cancer, providing us novel insights and avenues for searching potential biomarkers and therapeutic targets for breast cancer diagnosis and treatment.

## RESULTS

### The TUSC8 is significantly down-regulated in breast cancer tissues and predicts better prognosis of breast cancer patients

**Figure 1:**
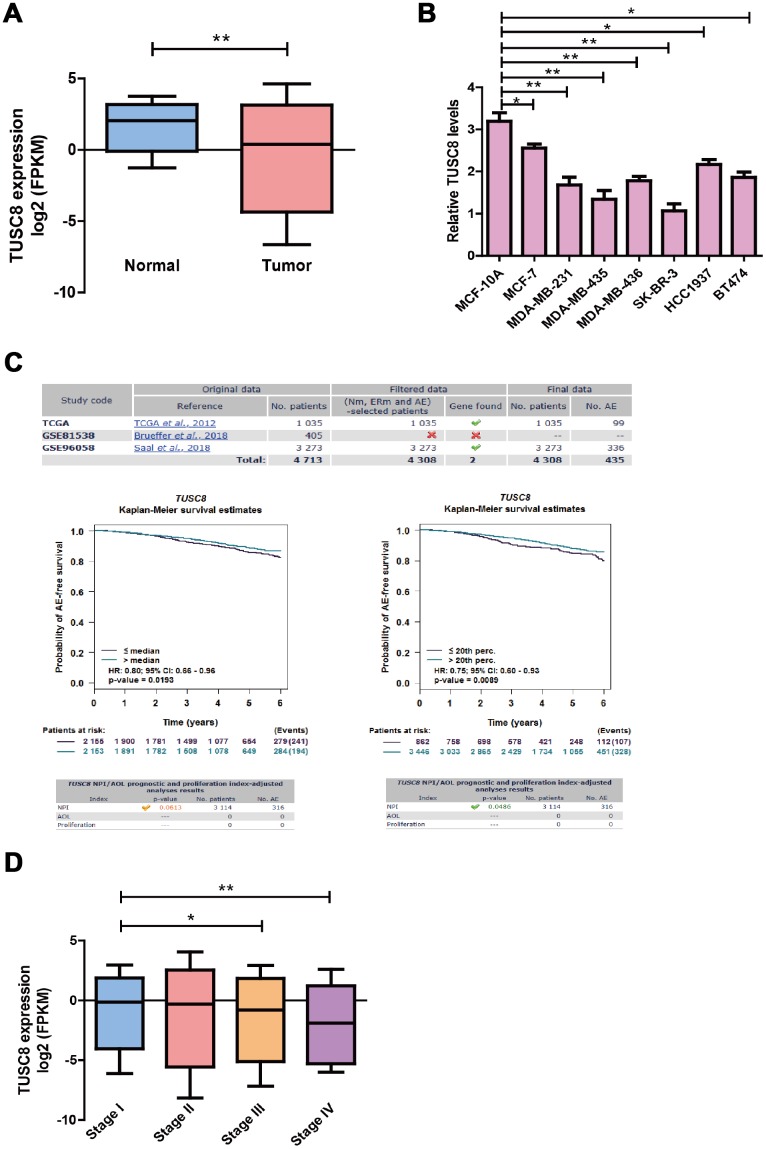
**The TUSC8 is significantly down-regulated in breast cancer tissues and predicts better prognosis of breast cancer patients.** (**A**) The down-regulation of TUSC8 expression in breast cancer samples (n=1104) compared with adjacent normal breast tissues (n=113) in TCGA database. (**B**) The TUSC8 expression levels were significantly down-regulated in multiple breast cancer cell lines compared with normal breast cancer cell line MCF-10A. (**C**) The survival curves of TUSC8 in breast cancer TCGA and GSE96058 dataset by using the median cut-off method and optimal cut-off method (20^th^ percentage). (**D**) The expression levels of TUSC8 in different stages of breast cancer patients. The asterisks (*, **) indicate a significant difference (*p* < 0.05, *p* < 0.01) respectively. Abbreviations: NPI: Nottingham prognostic index; AOL: Adjuvant! Online; AE: Any event; Nm: Nodal status mixed (contain positive and negative); ERm: Oestrogen receptor status mixed (contain positive and negative).

To identify the expression pattern of TUSC8 in breast cancer progression, we observed the significant down-regulation of TUSC8 expression in breast cancer samples (n=1104) compared with adjacent normal breast tissues (n=113) in TCGA database (*p* < 0.01) (Figure 1A). For the TUSC8 expression levels in breast cancer cell lines, the data indicated that TUSC8 expressions were significantly down-regulated in multiple breast cancer cell lines compared with normal breast cancer cell line MCF-10A by RT-PCR assay (*p* < 0.05, *p* < 0.01 respectively) (Figure 1B). Moreover, for the overall survival and prognosis value of TUSC8 in breast cancer patients, the curve demonstrated that the high expression of TUSC8 showed better overall survival of breast cancer patients compared with low expression group in TCGA database and GSE dataset (Figure 1C). Additionally, for the expression levels of TUSC8 in different stages of breast cancer patients in TCGA cohort, the results suggested that TUSC8 expression reduced gradually from stage I to stage IV (*p* < 0.05, *p* < 0.01 respectively) (Figure 1D), indicating that TUSC8 was associated with breast cancer progression.

### TUSC8 inhibits breast cancer cell growth, invasion and metastasis through regulating the expression of epithelial–mesenchymal transition (EMT) related markers

**Figure 2:**
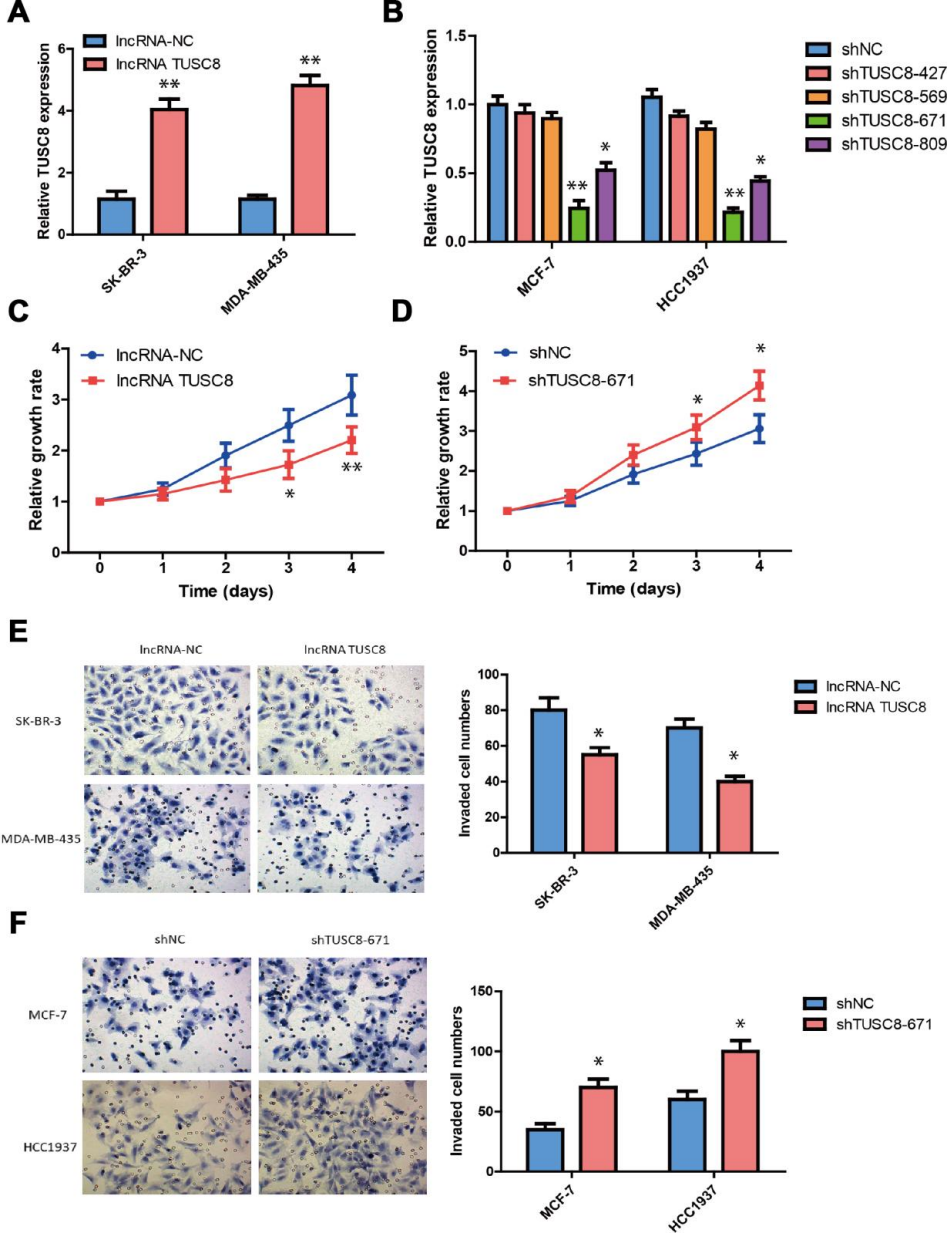
**TUSC8 inhibits breast cancer cell growth, invasion and metastasis.** (**A**) The good over-expression efficiency of TUSC8 in breast cancer cell lines SK-BR-3 and MDA-MB-435. (**B**) The good knock-down efficiency of TUSC8 in breast cancer cell lines MCF-7 and HCC1937 by using different shRNA subclones. (**C**) Over-expression of TUSC8 significantly suppressed breast cancer cell growth by cell proliferation assay. (**D**) Knock-down of TUSC8 drastically promoted breast cancer cell growth by cell proliferation assay. (**E**) Over-expression of TUSC8 reduced the cell invasive capacities in breast cancer cell lines SK-BR-3 and MDA-MB-435 by transwell invasion assay. (**F**) Knock-down of TUSC8 enhanced the cell invasive capacities in breast cancer cell lines MCF-7 and HCC1937 by transwell invasion assay. The asterisks (*, **) indicate a significant difference (*p* < 0.05, *p* < 0.01) respectively.

To explore the biological functions of TUSC8 in breast cancer development, we established the stable TUSC8 over-expression and knock-down cell lines. Lentiviruses for TUSC8 over-expression and negative control lentiviruses (NC) were used to infect the SK-BR-3 and MDA-MB-435 cells, whose TUSC8 expression were relatively lower among cell line panels. Similarly, lentiviruses expressing specific shRNAs targeting TUSC8 (shTUSC8) and negative control shRNA (shNC) were used in MCF-7 and HCC1937 cells, whose TUSC8 expression were relatively higher among cell line panels. The good over-expression or knock-down efficiency of TUSC8 in these cell lines were confirmed by RT-PCR assay (*p* < 0.05, *p* < 0.01 respectively) (Figure 2A, 2B). In the next step, the effect of TUSC8 on cell growth and proliferation was evaluated by CCK-8 assays and the results indicated that over-expression of TUSC8 decreased the growth rate of SK-BR-3 cells compared with the negative control group (*p* < 0.05, *p* < 0.01 respectively) (Figure 2C), while knock-down of TUSC8 increased the cellular growth rate in MCF-7 cells compared with the negative control group (*p* < 0.05) (Figure 2D). Furthermore, the transwell invasion assay was utilized to assess the effect of TUSC8 on cell invasion and metastasis. The results showed that TUSC8 over-expression reduced the cell invasive capacities in breast cancer cell lines SK-BR-3 and MDA-MB-435 (*p* < 0.05) (Figure 2E), whereas TUSC8 inhibition enhanced the cell invasive capacities in breast cancer cell lines MCF-7 and HCC1937 (*p* < 0.05) (Figure 2F).

**Figure 3:**
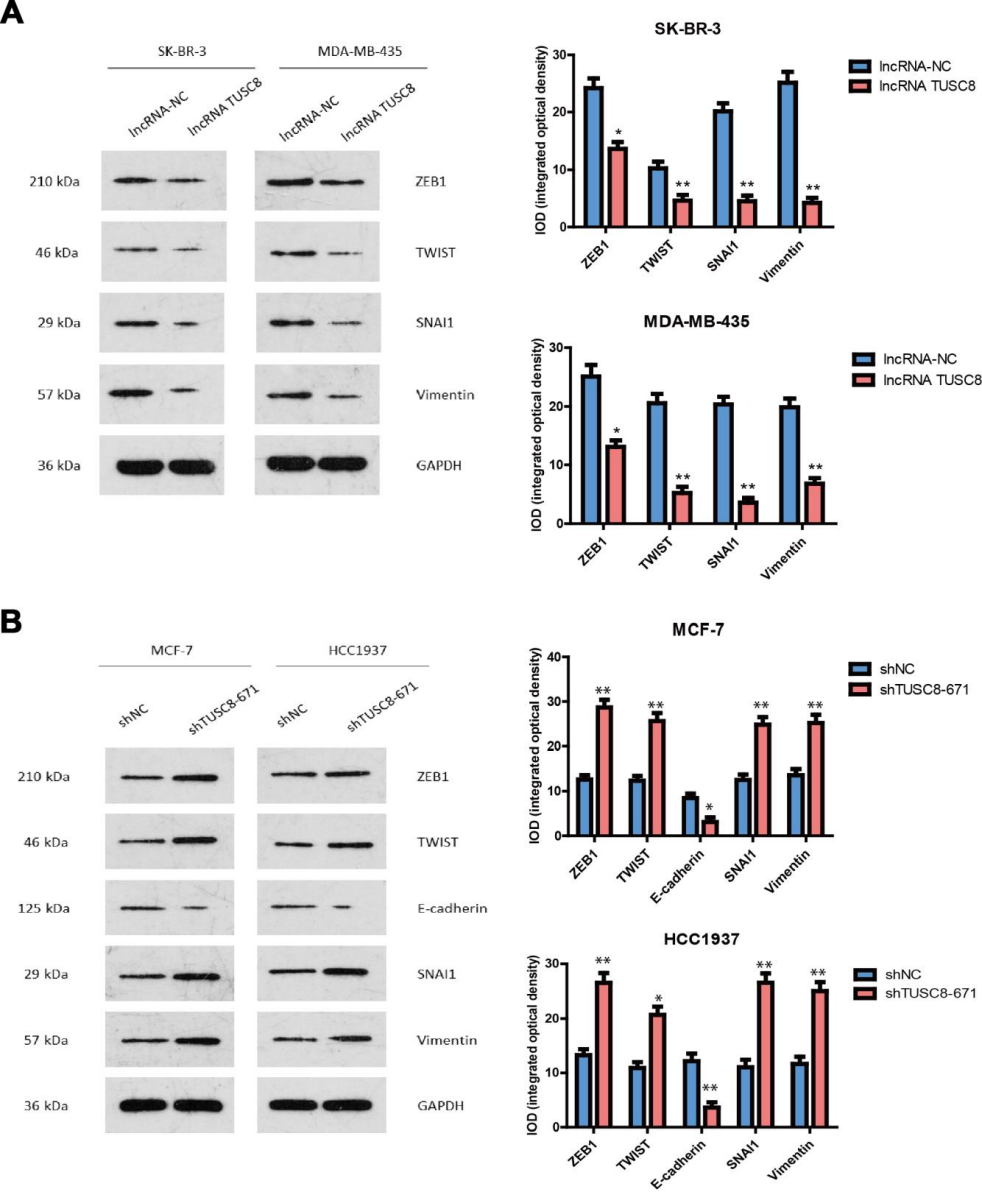
**TUSC8 affects breast cancer cell metastasis through regulating the expression of epithelial–mesenchymal transition (EMT) related markers.** (**A**) Over-expression of TUSC8 down-regulated the expression of mesenchymal related markers (ZEB1, TWIST, SNAI1 and Vimentin) in breast cancer cell lines SK-BR-3 and MDA-MB-435 by western blot. (**B**) Knock-down of TUSC8 up-regulated the expression of mesenchymal related markers (ZEB1, TWIST, SNAI1 and Vimentin), while down-regulated the expression of epithelial related marker (E-cadherin) in breast cancer cell lines MCF-7 and HCC1937 by western blot. The asterisks (*, **) indicate a significant difference (*p* < 0.05, *p* < 0.01) respectively.

Epithelial–mesenchymal transition (EMT) is an essential process in cancer metastasis, which makes the cell morphology change from epithelial to mesenchymal-like, so as to facilitate cell migration and invasion. Therefore, in this study we investigated whether EMT is a key factor for TUSC8-mediated cancer metastasis and checked various EMT related markers expression in breast cancer cell models. The western blot and the band intensity analysis hinted that over-expression of TUSC8 down-regulated the expression of mesenchymal related markers (ZEB1, TWIST, SNAI1 and Vimentin) in breast cancer cell lines SK-BR-3 and MDA-MB-435 (*p* < 0.05, *p* < 0.01 respectively) (Figure 3A). Conversely, knock-down of TUSC8 up-regulated the expression of mesenchymal related markers (ZEB1, TWIST, SNAI1 and Vimentin), and down-regulated the expression of epithelial related marker (E-cadherin) in breast cancer cell lines MCF-7 and HCC1937 (*p* < 0.05, *p* < 0.01 respectively) (Figure 3B).

### TUSC8 knock-down promotes the tumorigenicity of breast cancer cells *in vivo*

**Figure 4:**
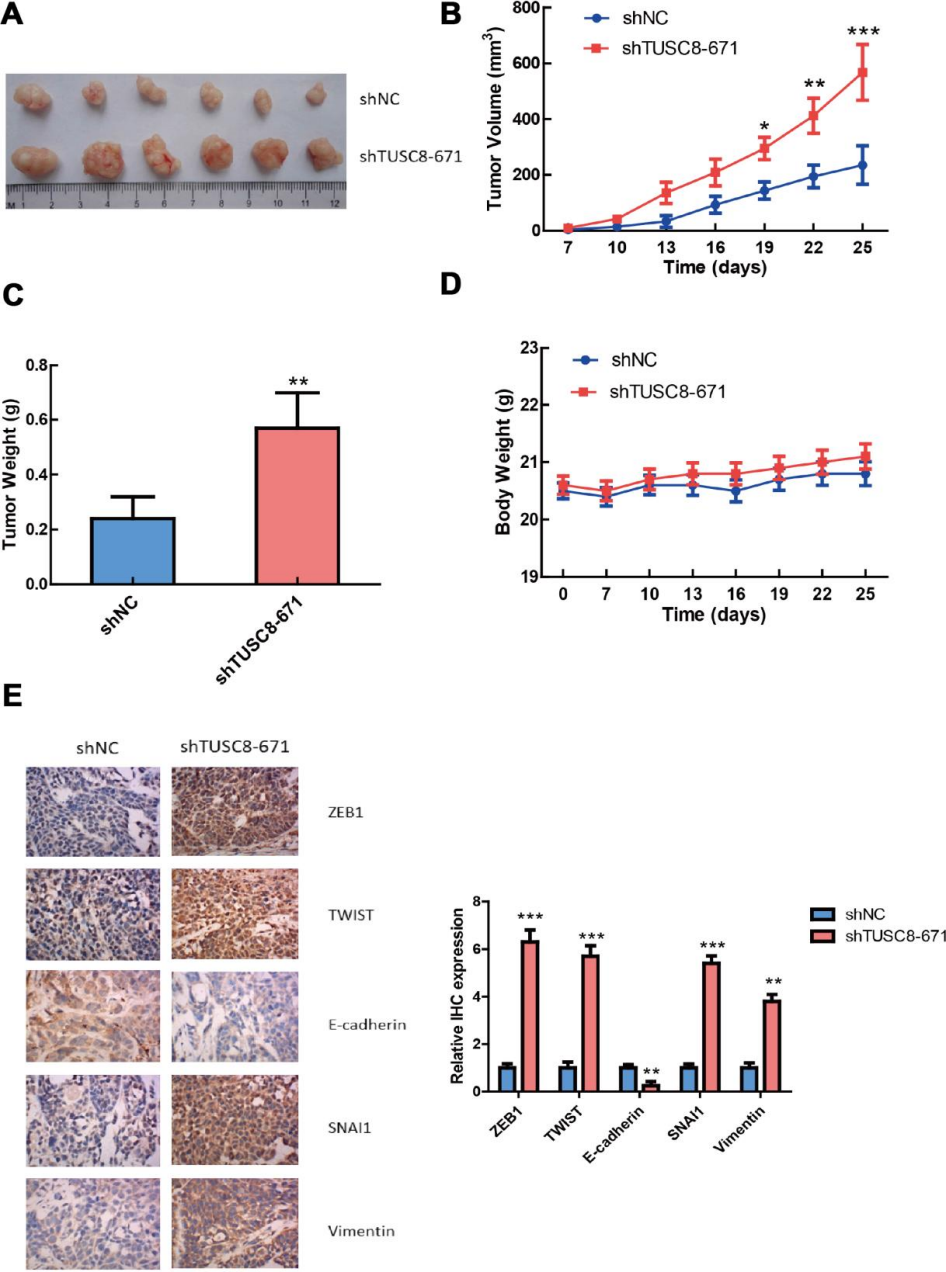
**TUSC8 knock-down promotes tumorigenicity and tumor growth of breast cancer cells *in vivo*.** (**A**, **B**) Stably knock-down of TUSC8 promoted breast cancer cell growth and increased tumor volume in MCF-7 cell line when subcutaneously injection into nude mice. (**C**) Knock-down of TUSC8 significantly increased the average tumor weight of breast tumors formed in the nude mice. (**D**) The body weight of nude mice exhibited no significant difference between TUSC8 knock-down group and negative control group. (**E**) The IHC staining of EMT related markers in nude mice breast tumors of TUSC8 knock-down group and negative control group. The asterisks (*, **, ***) indicate a significant difference (*p* < 0.05, *p* < 0.01, *p* < 0.001) respectively.

To further validate the functional role of TUSC8 *in vivo*, we adopted a xenograft mouse model through subcutaneously injecting negative control (NC) and TUSC8 knockdown MCF-7 cells into nude mice. The results showed that tumors grown from TUSC8 stable knockdown MCF-7 cells were much bigger and had larger tumor volumes than tumors grown from control cells (*p* < 0.05, *p* < 0.01, *p* < 0.001 respectively) (Figure 4A, 4B). The average tumor weight in the shTUSC8 group was also much higher than that in the control group (*p* < 0.01) (Figure 4C). However, the body weight of nude mice exhibited no significant difference between the TUSC8 knockdown group and the control group (Figure 4D). Furthermore, the IHC staining of EMT related markers in tumor tissues indicated that the expression of mesenchymal markers (including ZEB1, TWIST, SNAI1 and Vimentin) were significantly up-regulated and the epithelial marker E-cadherin expression was down-regulated in TUSC8 knockdown group compared with the control group (*p* < 0.01, *p* < 0.001 respectively) (Figure 4E).

### TUSC8 acts as a molecular sponge for miR-190b-5p in breast cancer cells

**Figure 5:**
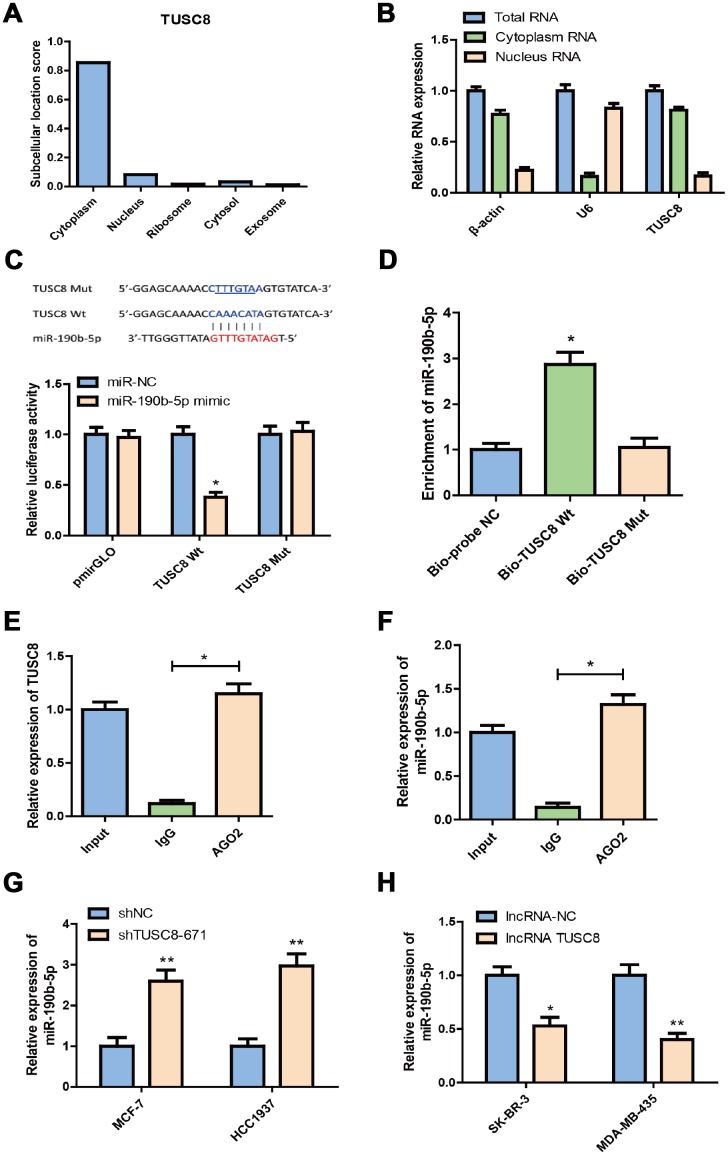
**TUSC8 functions as molecular sponge for miR-190b-5p in breast cancer cells.** (**A**) The subcellular location score of TUSC8 in IncLocator prediction database. (**B**) The relative TUSC8 expression levels in the cytoplasm and nucleus of MCF-7 cells. (**C**) Complementary sequence between miR-190b-5p and wild type (wt) TUSC8. The putative binding sites of miR-190b-5p were mutated in mutant (mut) TUSC8. MCF-7 cells that were co-transfected with miR-190b-5p mimics and wt or mut TUSC8 vectors were measured for luciferase activity. (**D**) miR-190b-5p was highly enriched in the sample pulled down with biotinylated wt TUSC8 rather than mut TUSC8 by RNA pull-down assay. (**E**, **F**) AGO2-RIP assay was performed in SK-BR-3 cell lysates, followed by qRT-PCR to detect TUSC8 and miR-190b-5p association with AGO2. The results indicated that TUSC8, miR-190b-5p and AGO2 formed a complex in SK-BR-3 cells. (**G**) Knock-down of TUSC8 significantly elevated the miR-190b-5p expression level in MCF-7 and HCC1937 cell lines. (**H**) Over-expression of TUSC8 significantly reduced the miR-190b-5p expression level in SK-BR-3 and MDA-MB-435 cell lines. The asterisks (*, **) indicate a significant difference (*p* < 0.05, *p* < 0.01) respectively.

To investigate the underlying mechanisms of TUSC8 in breast cancer, we first checked the subcellular location of TUSC8 in breast cancer cells by using IncLocator prediction database and RT-PCR assays for cytoplasm and nucleus RNA expression. The results suggested that the subcellular location score of TUSC8 was mainly enriched in cytoplasm as compared with nucleus, ribosome, cytosol and exosome (Figure 5A). And the relative expression level of TUSC8 was much higher in the cytoplasm than that in the nucleus of MCF-7 cells (Figure 5B). In the following step, we used the starBase v3.0 database to explore the association between TUSC8 and miRNAs, and we found that miR-190b-5p was predicted to interact with TUSC8 (Figure 5C). In order to test the binding sites of miR-190b-5p in TUSC8, we subcloned the wild-type TUSC8 transcript or its mutant sequence (the mutant binding sites of miR-190b-5p) into a luciferase reporter construct, and then transiently co-transfected the reporter constructs with miR-190b-5p mimics or negative control mimics (miR-NC) into MCF-7 cells. The results demonstrated that miR-190b-5p significantly reduced the luciferase activities in MCF-7 cells transfected with the wild-type TUSC8 construct, but did not affect the luciferase activity in the mutant TUSC8 construct (*p* < 0.05) (Figure 5C).

To further confirm the direct association between TUSC8 and miR-190b-5p, the RNA pull-down assay and AGO2-RIP assay were carried out. The data of RNA pull-down assay showed that compared with the treatment of Bio-probe NC, the treatment of Bio-TUSC8 Wt significantly increased enrichment of miR-190b-5p (*p* < 0.05), while no significant change was found in enrichment of miR-190b-5p following the treatment of Bio-TUSC8 Mut (Figure 5D). Moreover, since AGO2 is a critical component of the RNA-induced silencing complex (RISC) and acts as a critical modulator for miRNA post-transcriptional repression, AGO2-RIP assay was performed in SK-BR-3 cell lysates, followed by RT-PCR to detect TUSC8 and miR-190b-5p association with AGO2. The results suggested that endogenous TUSC8 pull-down by AGO2 was significantly enriched in SK-BR-3 cells (*p* < 0.05), indicating that TUSC8, miR-190b-5p and AGO2 formed in the same RISC complex (Figure 5E, 5F). Additionally, knock-down of TUSC8 significantly elevated the miR-190b-5p expression level in MCF-7 and HCC1937 cell lines (*p* < 0.01) (Figure 5G), whereas over-expression of TUSC8 drastically reduced the miR-190b-5p expression level in SK-BR-3 and MDA-MB-435 cell lines (*p* < 0.05, *p* < 0.01 respectively) (Figure 5H). Together, these data demonstrated that TUSC8 could physically bind with miR-190b-5p and competitively sponge miR-190b-5p as a ceRNA to regulate its steady state.

### MYLIP is a direct target of miR-190b-5p in breast cancer cells

**Figure 6:**
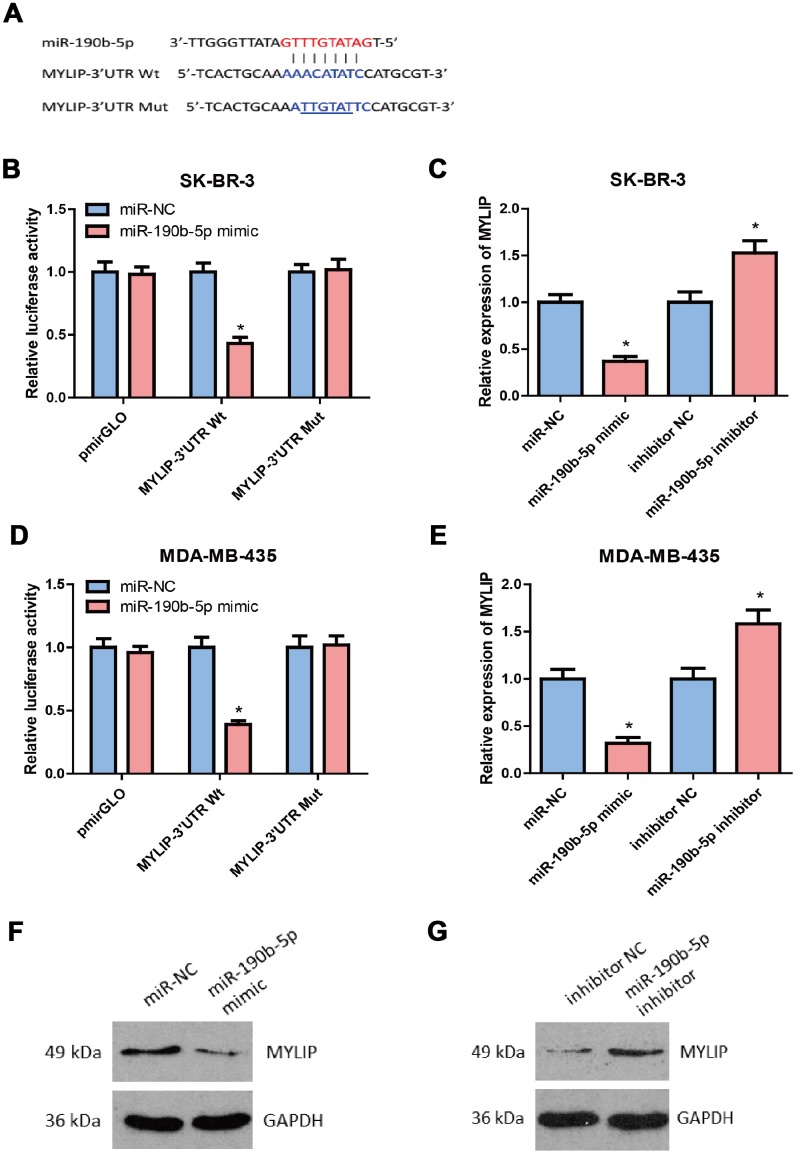
**MYLIP is a direct target of miR-190b-5p in breast cancer cells.** (**A**) Complementary sequence between miR-190b-5p and wild type (wt) 3′UTR of MYLIP. The putative binding sites of miR-190b-5p was mutated in 3′UTR of MYLIP (mut MYLIP-3′UTR). (**B**) SK-BR-3 cells that were co-transfected with miR-190b-5p mimics and wt or mut 3′UTR of MYLIP were subjected to luciferase activity measurement. (**C**) SK-BR-3 cells that were transfected with miR-190b-5p mimics or miR-190b-5p inhibitors were subjected to qRT-PCR for MYLIP mRNA expression. (**D**) MDA-MB-435 cells that were co-transfected with miR-190b-5p mimics and wt or mut 3′UTR of MYLIP were subjected to luciferase activity measurement. (**E**) MDA-MB-435 cells that were transfected with miR-190b-5p mimics or miR-190b-5p inhibitors were subjected to qRT-PCR for MYLIP mRNA expression. (**F**) miR-190b-5p overexpression reduced the protein expression level of MYLIP in MDA-MB-435 cells. (**G**) miR-190b-5p inhibition increased the protein expression level of MYLIP in SK-BR-3 cells. The asterisk (*) indicates a significant difference (*p* < 0.05).

By using different bioinformatics tools (including TargetScan, PicTar, PITA, miRanada and starBase v3.0) to predict the putative downstream targets of miR-190b-5p, we found the candidate target MYLIP overlapped in these databases containing the complementary binding sites for the seed region of miR- 190b-5p (Figure 6A). Then, we constructed the MYLIP 3′-UTR Wt (wild type) and Mut (mutant) vectors according to their binding sites with miR-190b-5p seed sequences (highlighted in red) (Figure 6A), and co-transfected them with miR-190b-5p mimic or mimic control in SK-BR-3 and MDA-MB-435 cells for dual luciferase reporter assay. The data hinted that miR-190b-5p over-expression markedly decreased the relative luciferase activity in MYLIP 3’-UTR Wt group rather than MYLIP 3’-UTR Mut group (*p* < 0.05) (Figure 6B, 6D), which suggested that miR-190b-5p could directly regulate the expression of MYLIP through targeting its 3’-UTR region. Next, we detected the mRNA and protein expression levels of MYLIP in SK-BR-3 and MDA-MB-435 cell lines after transfected with miR-190b-5p mimic, miR-190b-5p inhibitor and their negative controls. The results demonstrated that over-expression of miR-190b-5p could significantly down-regulate the MYLIP mRNA and protein expression levels (*p* < 0.05) (Figure 6C, 6E, 6F). Conversely, inhibition of miR-190b-5p expression could significantly up-regulate the MYLIP mRNA and protein expression levels (*p* < 0.05) (Figure 6C, 6E, 6G). All of the above data indicated that MYLIP was a direct downstream target of miR-190b-5p in breast cancer cells.

### TUSC8 inhibits breast cancer metastasis partly through miR-190b-5p-MYLIP axis

**Figure 7:**
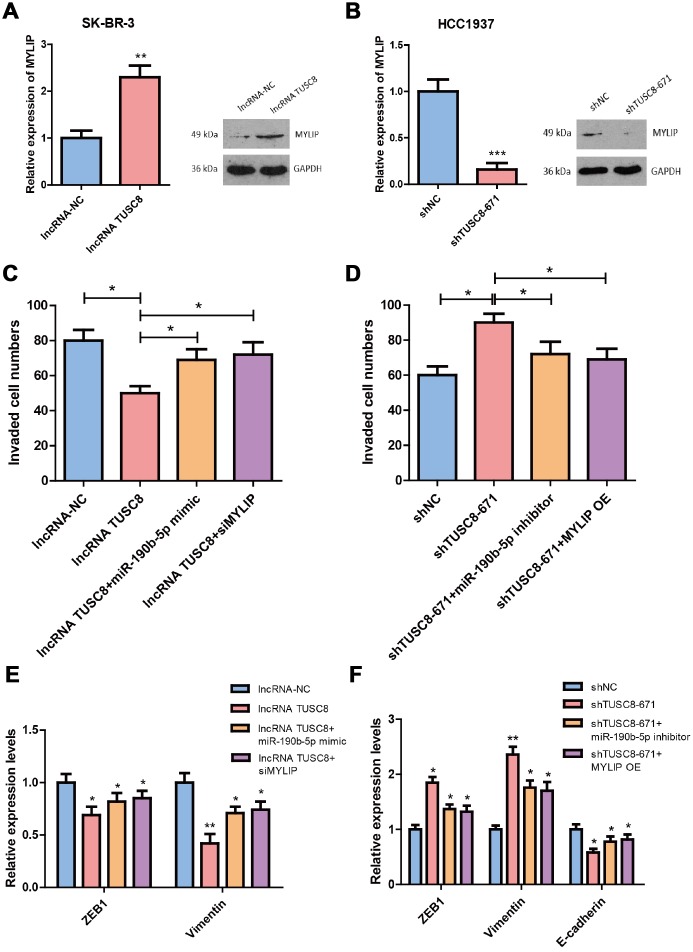
**TUSC8 inhibits breast cancer cell invasive capacity partly through miR-190b-5p-MYLIP axis.** (**A**) Over-expression of TUSC8 significantly increased the mRNA and protein levels of MYLIP in SK-BR-3 cells. (**B**) Knock-down of TUSC8 drastically suppressed the mRNA and protein levels of MYLIP in HCC1937 cells. (**C**) Over-expression of miR-190b-5p or inhibition of MYLIP partly abolished the reduced cell invasive capacities mediated by TUSC8 over-expression in SK-BR-3 cells. (**D**) Inhibition of miR-190b-5p or MYLIP over-expression partly rescued the enhanced cell invasive capacities mediated by TUSC8 knock-down in HCC1937 cells. (**E**) Over-expression of miR-190b-5p or inhibition of MYLIP partly abolished the EMT related markers expression mediated by TUSC8 over-expression in SK-BR-3 cells. (**F**) Inhibition of miR-190b-5p or MYLIP over-expression partly rescued the EMT related markers expression mediated by TUSC8 knock-down in HCC1937 cells. The asterisks (*, **, ***) indicate a significant difference (*p* < 0.05, *p* < 0.01, *p* < 0.001) respectively.

We then explored whether TUSC8 regulates breast cancer metastasis via miR-190b-5p-MYLIP axis. Using RT-PCR assay and western blot, we confirmed that over-expression of TUSC8 significantly increased the mRNA and protein levels of MYLIP in SK-BR-3 cells (*p* < 0.01) (Figure 7A), and knock-down of TUSC8 drastically suppressed the mRNA and protein levels of MYLIP in HCC1937 cells (*p* < 0.001) (Figure 7B). These data suggested that MYLIP expression was positively correlated with TUSC8 expression in breast cancer. Additionally, over-expression of TUSC8 reduced the cell invasive capacities and affected EMT related markers expression (down-regulation of ZEB1 and Vimentin) in SK-BR-3 cells. Ectopic expression of miR-190b-5p or MYLIP inhibition partly abolished these effects (*p* < 0.05, *p* < 0.01 respectively) (Figure 7C, 7E). Inversely, knock-down of TUSC8 enhanced the cell invasive capacities and affected EMT related markers expression (up-regulation of ZEB1/Vimentin, and down-regulation of E-cadherin) in HCC1937 cells, which were partially rescued by inhibition of miR-190b-5p or MYLIP over-expression (*p* < 0.05, *p* < 0.01 respectively) (Figure 7D, 7F).

### Clinical relevance and diagnostic value of TUSC8 and MYLIP in breast cancer patients

**Figure 8:**
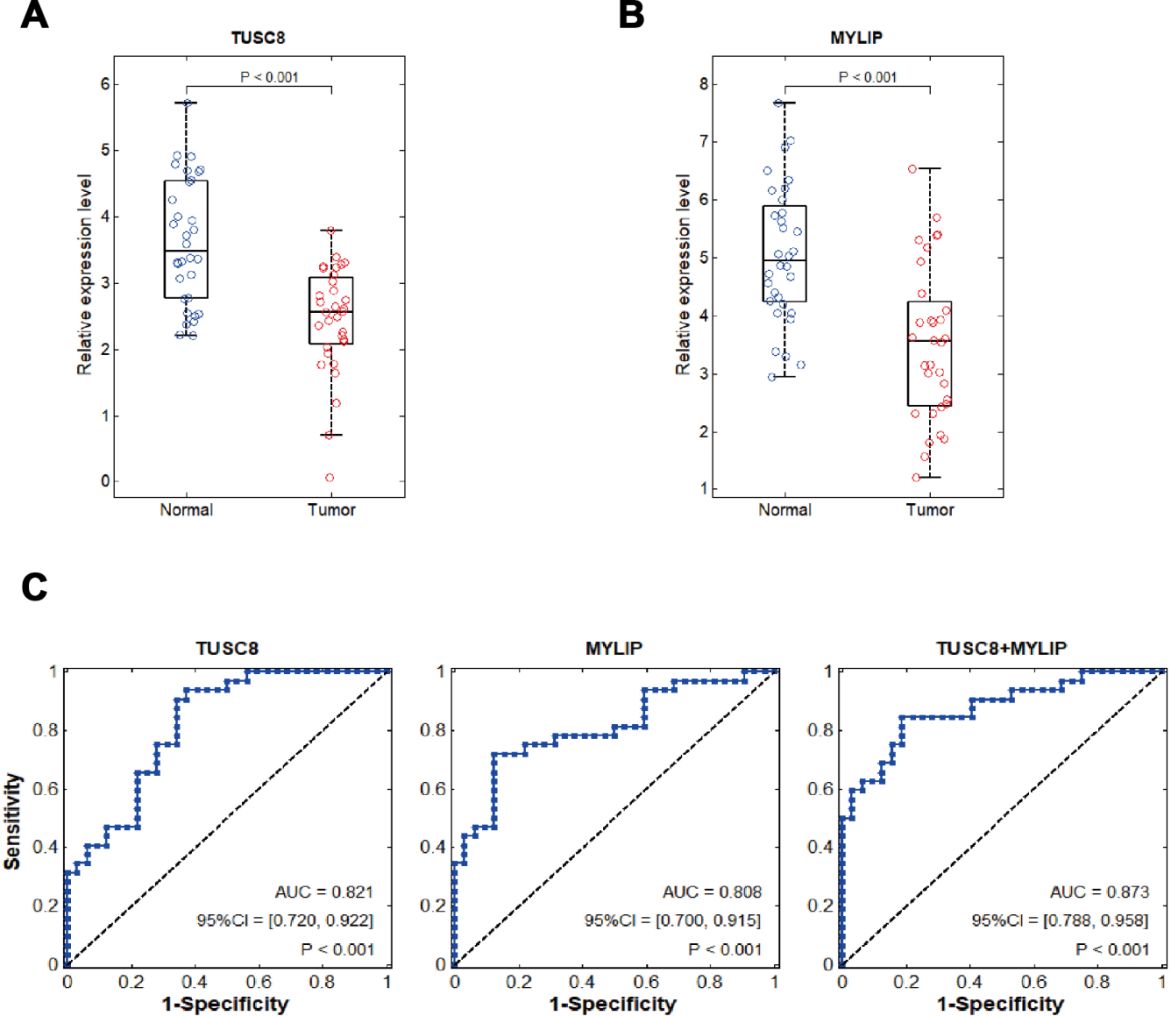
**Clinical diagnostic value of TUSC8 and MYLIP in breast cancer patients.** (**A**, **B**) The relative expression levels of TUSC8 and MYLIP in breast cancer tissues (n=32) and adjacent normal breast tissues (n=32). (**C**) The receiver operating characteristic curves (ROC) and area under ROC curves (AUC) displaying the sensitivity and specificity of TUSC8, MYLIP and their combination for the diagnosis of breast cancer.

Using RT-PCR assay in tumorous and adjacent normal breast tissues in 32 cases of breast cancer patients, we identified that TUSC8 and MYLIP were significantly down-regulated in breast cancer (*p* < 0.001) (Figure 8A, 8B). In the following step, we verified TUSC8 down-regulation in an expanded breast cancer sample cohort and conducted the clinicopathological correlation analysis using median cut-off method. The results revealed that low expression of TUSC8 was significantly associated with aggressive tumor behavior in large tumor size (*p*=0.0009), tumor encapsulation (*p*=0.005), venous invasion (*p*=0.002) and advanced TNM staging (*p*=0.002) of breast cancer patients (Table 1). In order to further evaluate the diagnostic values of TUSC8 and MYLIP in breast cancer, we used the receiver operating characteristic curve (ROC) analyses to assess the potential use of these two biomarkers for differentiation of breast cancer patients. As depicted in Figure 8C, the area under the curve (AUC) of TUSC8 for the diagnosis was 0.821 with a 95% confidence interval (CI) of 0.720-0.922 (*p* < 0.001), the AUC of MYLIP for the diagnosis was 0.808 with a 95% CI of 0.700-0.915 (*p* < 0.001), and the AUC of TUSC8+MYLIP for the diagnosis was 0.873 with a 95% CI of 0.788-0.958 (*p* < 0.001). These results hinted that the combination usage of TUSC8 and MYLIP might serve as promising diagnostic biomarkers for breast cancer.

**Table 1:** The correlation between TUSC8 expression and clinicopathological characteristics of breast cancer patients.

**Parameters**	**TUSC8 expression**				P *
	**High (N=32)**		**Low (N=32)**		
Age (years)					0.089
>60	12	(18.8%)	19	(29.7%)	
<=60	20	(31.2%)	13	(20.3%)	
Tumor size					0.0009*
>5cm	10	(15.6%)	24	(37.5%)	
<=5cm	22	(34.4%)	8	(12.5%)	
Molecular subtype					0.489
Luminal A/B	11	(17.2%)	8	(12.5%)	
HER2+	12	(18.8%)	10	(15.6%)	
Basal-like	9	(14.1%)	14	(21.8%)	
Tumor encapsulation					0.005*
Presence	21	(32.8%)	9	(14.1%)	
Absence	11	(17.2%)	23	(35.9%)	
Venous invasion					0.002*
Presence	9	(14.1%)	22	(34.4%)	
Absence	23	(35.9%)	10	(15.6%)	
TNM staging					0.002*
I-II	20	(31.2%)	7	(10.9%)	
III-IV	12	(18.8%)	25	(39.1%)	

* Fisher exact test.

## DISCUSSION

Dysregulation of lncRNA has been closely related to the carcinogenesis and progression of multiple human cancers, and it also acts as an essential modulatory molecule in breast cancer development. In this study, we discovered the functional roles of a novel lncRNA TUSC8 in breast tumorigenesis for the first time and demonstrated that TUSC8 was significantly down-regulated in breast cancer tissues and its high expression predicted better prognosis of breast cancer patients. In addition, TUSC8 significantly suppressed the proliferation, invasion and metastasis of breast cancer cells *in vitro* and repressed tumorigenicity *in vivo*. Moreover, the clinicopathological correlation analysis revealed that low expression of TUSC8 was significantly associated with aggressive tumor behavior in large tumor size, tumor encapsulation, venous invasion and advanced TNM staging of breast cancer patients. And the ROC analysis suggested that TUSC8 might serve as a promising diagnostic biomarker for breast cancer.

Mounting evidences showed that lncRNAs localized in the cytoplasm could function as competing endogenous RNAs (ceRNA) and natural miRNA sponges to post-transcriptionally regulate gene expression through competitively binding common miRNAs [17–19]. For example, lncRNA n335586 promoted hepatocellular carcinoma cells migration and invasion through facilitating the expression of its host gene CKMT1A by competitively binding miR-924 [20]. GAS5 acted as a ceRNA to regulate hZIP1 by sponging miR-223 in the progression of clear cell renal cell carcinoma and targeting the GAS5/miR-223/hZIP1 axis served as a therapeutic strategy for patients [21]. LINC00511 functioned as a ceRNA for miR-185-3p to positively recover E2F1 protein. Furthermore, the transcription factor E2F1 bound with the promoter region of Nanog gene to promote its transcription and facilitate the breast cancer stemness and tumorigenesis [22].

In our previous studies, we revealed that inhibition of MYLIP facilitated the migration and metastasis of breast cancer cells, providing us a promising therapeutic target to interfere breast cancer development [23]. In this case, in order to further investigate the upstream regulatory ceRNA network of MYLIP, we launched this study in breast cancer. As lncRNA TUSC8 was relatively rare studied in cancer development process, it had been reported to inhibit metastasis of cervical cancer cells. The biological functions and underlying molecular mechanisms of TUSC8 with respect to breast cancer remain largely unclear, suggesting that TUSC8 might be a novel research field for breast tumorigenesis. Based on the above two reasons, we first explored the potential miRNAs which could directly bind to the MYLIP 3′-UTR region through bioinformatics tools (miRecords, miRanda, TargetScan, PITA, RNAhybrid and starBase), and narrowed down the list through checking the binding sequences of miRNAs with TUSC8. Then we got three miRNA candidates (miR-190b-5p, miR-92b, miR-222), which possessed seven continuous base pair match with TUSC8 sequence. In the following step, we checked the expression levels of these three miRNA candidates in eight breast cancer cell lines and compared their expression tendency with TUSC8 and MYLIP in the same breast cancer cell lines. Finally, we found that miR-190b-5p was a more suitable candidate which showed the opposite expression pattern with both TUSC8 and MYLIP in breast cancer cells. Furthermore, through literature searching, miR-190b was reported to be significantly up-regulated in breast tumors and contributed to hormone-dependent breast carcinogenesis [24, 25]. Therefore, we constructed the ceRNA network of TUSC8, miR-190b-5p and MYLIP in breast cancer development.

Additionally, in our study, the results of luciferase reporter, RIP and RNA pull-down assays proved that miR-190b-5p was a direct target of TUSC8. Moreover, our data showed that TUSC8 functioned as a ceRNA of MYLIP through competitively binding with miR-190b-5p. However, over-expression of miR-190b-5p or inhibition of MYLIP did not completely abolish the reduced cell invasive capacities mediated by TUSC8 over-expression, indicating that other mechanisms might be also involved in the regulation of this process.

The epithelial-mesenchymal transition (EMT) is a reversible process by which a polarized epithelial cell is transformed into a cell with a mesenchymal phenotype and a conserved cellular program that alters cell shape, adhesion and movement [26]. The shift to mesenchymal-like phenotype can promote tumor cell intravasation of surrounding blood vessels and migration to new organs [27]. In addition to motility, EMT is associated with enhanced stem cell properties and drug resistance, thus it can drive metastasis, tumor recurrence, and therapy resistance in the context of different cancers [28]. Numerous studies have already emphasized the crucial roles of lncRNAs in the EMT process of cancer development. LncRNA PTAR was reported to promote EMT and metastasis in serous ovarian cancer by competitively binding miR-101-3p to regulate ZEB1 expression [29]. LncRNA FEZF1-AS1 enhanced EMT through suppressing E-cadherin and regulating Wnt/beta-catenin signaling in non-small cell lung cancer (NSCLC). Moreover, down-regulation of lncRNA FEZF1-AS1 suppressed cell EMT process by increasing the expression of E-cadherin and ZO-1, whereas, decreasing the expression of Slug, Twist and Vimentin in NSCLC cells [30]. In this research, we demonstrated that knock-down of TUSC8 facilitated EMT process through up-regulating the expression of mesenchymal related markers (ZEB1, Twist, SNAI1 and Vimentin), while down-regulating the expression of epithelial related marker (E-cadherin) in breast cancer cell and mice models, thus contributing to the metastasis of breast cancer.

Myosin regulatory light chain interacting protein (MYLIP) belongs to the cytoskeletal protein clusters and is involved in the regulation of cell movement and migration through interacting the cell membrane proteins with myosin cytoskeleton. It mainly plays essential roles in the modulation of cell motility, the remodeling of cytoskeletal proteins, and the interaction with extracellular matrix [23]. The research conducted in prostate cancer (PC) suggested that CNPY2 promoted cell growth of PC cells by inhibition of androgen receptor (AR) protein degradation through MYLIP-mediated AR ubiquitination. And CNPY2 decreased the ubiquitination activity of MYLIP by inhibition of interaction between MYLIP and UBE2D1, an E2 ubiquitin ligase [31]. In this study, we validated that MYLIP was a direct target of miR-190b-5p in breast cancer cells and TUSC8 inhibited breast cancer metastasis partly through miR-190b-5p/MYLIP axis. For the clinical relevance and diagnostic value in breast cancer patients, the ROC analyses indicated that the combination usage of TUSC8 and MYLIP might become novel promising biomarkers and targets for breast cancer diagnosis and treatment.

## MATERIALS AND METHODS

### Human tissue specimens

All the human breast cancer tissues and their paired adjacent normal breast tissues were collected during surgical resection from the Department of Oncology, Xiangya Hospital. Diagnosis was based on pathological evidence, and collected tissue specimens were immediately snap frozen in liquid nitrogen and stored at -80°C until used. The procedure of present study had been approved by the Institutional Ethics Review Committee of Xiangya Hospital. Written informed consent had been obtained from all participants.

### Cell culture

The MCF-10A cell line was cultured in DMEM and Ham's F12 base with 20ng/ml epidermal growth factor (Sigma, E-9644), 100ng/ml cholera toxin (Sigma, C-8052), 0.01mg/ml human insulin (Sigma, I-2643), 500ng/ml hydrocortisone (Sigma, H-0888), 5% Chelex-treated horse serum, and 1% antibiotics. The MDA-MB-435 and MDA-MB-436 cell lines were cultured in ATCC-formulated Leibovitz's L-15 Medium base with 0.01mg/ml bovine insulin, 0.01mg/ml glutathione, 10% fetal bovine serum (FBS) and 1% antibiotics. The BT-474 and HCC1937 cell lines were cultured in RPMI-1640 with 10% fetal bovine serum (FBS) and 1% antibiotics. The MCF-7, SK-BR-3 and MDA-MB-231 cell lines were cultured in DMEM with 10% fetal bovine serum (FBS) and 1% antibiotics. The cell lines were grown in a humidified incubator at 37°C with 5% CO_2_.

### Cell transfection

The miR-190b-5p mimic, miR-190b-5p inhibitor and corresponding negative control (miR-NC, inhibitor NC) were purchased from GenePharma (Shanghai, China). The MYLIP siRNA (siMYLIP) and corresponding negative control siRNA (siNC) were also purchased from GenePharma (Shanghai, China). The transfections were conducted using Lipofectamine 2000 (Invitrogen, Carlsbad, USA) following the manufacturer's protocol.

### Over-expression or knock-down of TUSC8

For over-expression of TUSC8, the cDNA encoding TUSC8 was PCR amplified and subcloned into the lentiviral vector pLV (Addgene). Lentiviral preparations were generated by transient transfection of HEK293FT cells by using pLV-TUSC8, psPAX2 and pMD2.G plasmids. For knock-down of TUSC8, different TUSC8 shRNAs were inserted into lentiviral vector pLKO.1 (Addgene). Lentiviral preparations were generated the same as above. Lentiviruses were harvested at 48hrs after transfection and then filtered. Breast cancer cells were infected with above lentiviruses in the presence of 8μg/ml polybrene (Sigma-Aldrich). 48hrs after infection, the stable over-expression or knock-down cells were selected with puromycin (2μg/ml) for 1 week.

### miRNA and RNA extraction

Cellular miRNA extraction was performed using mirVana miRNA Isolation Kit (AM1560, Ambion) following the manufacturer’s instructions. Total RNA extraction was performed using TRIzol Reagent (Invitrogen, Carlsbad, CA, USA) following the manufacturer’s instructions.

### Quantitative real-time PCR assays

The miR-190b-5p quantitative real-time PCR assays were performed using TaqMan® MicroRNA Assays (Applied Biosystems, USA), and U6 snRNA (Applied Biosystems, USA) was used as an internal control. For the TUSC8, MYLIP and EMT related markers quantitative real-time PCR assays, GAPDH was used as an internal control. RT-PCR assays were performed by SYBR green qPCR SuperMix (Applied Biosystems Life Technologies, Foster, CA, USA) in ABI prism 7500 sequence detection system (Applied Biosystems Life Technologies). The relative gene expression levels were calculated using the 2^-△△Ct^ method.

### Western blot analysis

The total cell lysates were separated by SDS-PAGE gel and followed by Western blot. The images were processed and the integrated optical densities (IOD) of the bands were analyzed by Image Lab 4.0 (Bio-Rad Laboratories, Inc.) software packages. The antibodies used in this experiment were as follows: MYLIP (ab74562, Abcam, USA), E-Cadherin (ab76055, Abcam, USA), ZEB1 (ab181451, Abcam, USA), TWIST (ab49254, Abcam, USA), SNAI1 (13099-1-AP, Proteintech, USA), Vimentin (ab92547, Abcam, USA) and GAPDH (sc-365062, Santa Cruz, USA).

### Cell proliferation assay

Cell proliferation was examined by Cell Counting Kit-8 (Dojindo, Kumamoto, Japan). 2×10^3^ cells in 100μl medium were seeded into a 96-well plate in quadruplicate. 10μl of Cell Counting Kit-8 (CCK-8) (Dojindo, Kumamoto, Japan) was added to each well at 24, 48, 72 and 96h time points after the cells were seeded. The absorbance was measured at a wavelength of 450nm using a microplate reader (Thermo-Fisher Scientific) after the incubation for 2h.

### Transwell invasion assay

For the cell invasion assay, 24-well transwell plates with 8.0-μm-pore Matrigel-coated invasion chambers (BD Biosciences) were used. 1×10^5^ cells were seeded into the upper chambers with serum-free medium. The lower chambers were filled with medium containing 20% FBS to act as chemo-attractant. Cells were then incubated in the incubator for 18-24hrs. After the incubation, the invaded cells were fixed with methanol for 30mins and stained with crystal violet. The cells that had invaded through the membrane to the lower surface were photographed and counted under a microscope in 10 random fields. All experiments were performed in triplicates.

### Tumor xenograft model

Six- to eight-week-old female BALB/c nude mice (purchased from Beijing Vital River Laboratory Animal Technology, Beijing, China) were used in this experiment and randomly divided into two groups for six mice each. After the ovariectomy was performed, the 0.72mg/60days slow release estradiol pellets (purchased from Innovative Research of America, FL, USA) were adopted to give the estrogen supplement for supporting the growth of MCF-7 cells in nude mice. The pellets were implanted subcutaneously into the dorsal flank of female BALB/c nude mice. Then the MCF-7 cells (1×10^6^ per injection) that were transfected with sh-TUSC8 and sh-negative control (sh-NC), respectively, were implanted into the right flank of the mice through subcutaneous injection. 7 days after the injection, the tumor volumes and body weights of BALB/c nude mice were measured once three days until the 25 days. Tumor growth was monitored over time using electronic calipers. Tumor volumes were calculated by the modified ellipsoidal formula: volume = 1/2(length × width^2^). Then the mice were euthanized and the tumors were separated to weigh their weights. After that the tumors were fixed with phosphate-buffered neutral formalin and embedded by paraffin for IHC staining. All animal procedures were performed in accordance with institutional guidelines. All animal experimental procedures were approved by the Animal Ethics Committee of Xiangya Hospital, Central South University.

### Immunohistochemistry (IHC)

The tissues were sectioned, treated with 3% H_2_O_2_, and then incubated in 5% goat antiserum. Non-serial tissue sections were incubated with the primary antibodies against E-Cadherin (1:200; Abcam, USA), ZEB1 (1:100; Abcam, USA), TWIST (1:200; Boster, China), SNAI1 (1:100; Proteintech, USA), Vimentin (1:500; Abcam, USA) overnight, and then with biotin-labeled secondary antibodies. Streptavidin-peroxidase complex was added, and the sections were stained with 3,3′-diaminobenzidine (Maixin Biotech, Fuzhou, China) prior to microscopy analyses. All sections were independently scored by three experienced pathologists. Scoring was based on the percentages of positive cells with different staining intensities.

### RNA pull-down assay

A DNA fragment containing the full-length TUSC8 sequence or TUSC8 mutant sequence was PCR amplified using T7 RNA polymerase (Roche, Basel, Switzerland). The resulting plasmid DNA was linearized using the restriction enzyme XhoI. Biotin-labeled RNA was reversely transcribed using Biotin RNA Labeling Mix (Roche) and T7 RNA polymerase (Roche). The products were treated with RNase-free DNase I (Takara, Japan) and purified with the RNeasy Mini Kit (Qiagen, MD, USA). The biotin-labeled RNA was isolated with Dynabeads M-280 Streptavidin (Invitrogen, CA, USA). The miR-190b-5p present in the pull-down complex was detected by RT-PCR analysis.

### RNA immunoprecipitation (RIP) assay

The RIP assay was conducted by using the EZ-Magna RIP Kit (Millipore, MA, USA) following the manufacturer’s instructions. Briefly, cells were lysed in RIP lysis buffer, and RNAs magnetic beads were conjugated with a human anti-AGO2 antibody (ab32381, Abcam, USA) or with a negative control normal mouse anti-IgG (Millipore, USA). Subsequently, RT-PCR assay was carried out to detect co-precipitated RNAs.

### Luciferase reporter assay

The fragments of 3′-UTR of MYLIP or TUSC8 containing putative miR-190b-5p binding sites or the mutant sequences were synthesized and inserted into the pmirGLO vector (Promega, WI, USA). The cells were co-transfected with these vectors and miR-190b-5p mimic or mimic control (miR-NC). The Dual-Luciferase Reporter Assay System (Promega, WI, USA) was used to measure the Firefly and Renilla luciferase activities. Renilla luciferase activity was used as the normalized control.

### Statistical analysis

All quantitative data were expressed as mean values ± S.D. of at least 3 independent experiments. Statistical analysis was performed using SPSS (version 20.0, IBM, USA) software and GraphPad Prism (version 6.0, San Diego, CA, USA) software. Significant differences between two groups were compared using the Student’s t-test, and comparisons among more than two groups were performed using analysis of variance (ANOVA). And the Levene's test or F-test was used to assess variance homogeneity before the t-test and ANOVA. The survival curves were plotted by Kaplan-Meier method, and the survival differences were compared using the log-rank test. *p-*values < 0.05 were considered to be statistically significant.
